# A Repurposing Approach for Uncovering the Anti-Tubercular Activity of FDA-Approved Drugs with Potential Multi-Targeting Profiles

**DOI:** 10.3390/molecules24234373

**Published:** 2019-11-29

**Authors:** Basem Battah, Giulia Chemi, Stefania Butini, Giuseppe Campiani, Simone Brogi, Giovanni Delogu, Sandra Gemma

**Affiliations:** 1 Fondazione Policlinico Universitario A. Gemelli, IRCCS Rome. Largo A. Gemelli 8, 00168 Rome, Italy; basem.battah.sc@hotmail.com; 2 Department of Biotechnology (DoE 2018-2022), Chemistry and Pharmacy (DoE 2018-2022), University of Siena, via Aldo Moro 2, 53100 Siena, Italy; giu.chemi@gmail.com (G.C.); stefania.butini@unisi.it (S.B.); campiani@unisi.it (G.C.); 3 Department of Pharmacy, University of Pisa, via Bonanno 6, 56126 Pisa, Italy; 4 Institute of Microbiology, Università Cattolica del Sacro Cuore, Roma–Largo F. Vito 1, 00168 Rome, Italy; giovanni.delogu@unicatt.it; 5 Mater Olbia Hospital, SS 125 Orientale Sarda, 07026 Olbia, Italy

**Keywords:** multi-targeting drugs, tuberculosis, computational methods, FDA-approved drugs, drug repurposing

## Abstract

Tuberculosis (TB) is one of the top 10 causes of death worldwide. This scenario is further complicated by the insurgence of multidrug-resistant (MDR) and extensively drug-resistant (XDR) TB. The identification of appropriate drugs with multi-target affinity profiles is considered to be a widely accepted strategy to overcome the rapid development of resistance. The aim of this study was to discover Food and Drug Administration (FDA)-approved drugs possessing antimycobacterial activity, potentially coupled to an effective multi-target profile. An integrated screening platform was implemented based on computational procedures (high-throughput docking techniques on the target enzymes peptide deformylase and Zmp1) and in vitro phenotypic screening assays using two models to evaluate the activity of the selected drugs against *Mycobacterium tuberculosis* (*Mtb*), namely, growth of *Mtb* H37Rv and of two clinical isolates in axenic media, and infection of peripheral blood mononuclear cells with *Mtb*. Starting from over 3000 FDA-approved drugs, we selected 29 marketed drugs for submission to biological evaluation. Out of 29 drugs selected, 20 showed antimycobacterial activity. Further characterization suggested that five drugs possessed promising profiles for further studies. Following a repurposing strategy, by combining computational and biological efforts, we identified marketed drugs with relevant antimycobacterial profiles.

## 1. Introduction

Tuberculosis (TB), a disease caused by infection with *Mycobacterium tuberculosis* (*M. tuberculosis*), is one of the top 10 causes of death worldwide, accounting for 1.7 million deaths and 10.0 million new cases in 2017 [1]. Chemotherapy of TB is still a challenging task due to several factors related both to the specific biology of *Mtb* and the need to prevent the emergence of drug resistance. These factors translate to long-lasting and complex treatment approaches [2]. In particular, multidrug-resistant (MDR) and extensively drug-resistant (XDR) TB are associated with high rates of treatment failure [3,4]. Although isoniazid is still an important first-line antitubercular drug, its activity against dormant bacilli is suboptimal, thus prompting the emergence of resistance if administered alone [5,6]. Among the approaches employed to tackle the problem of drug resistance in infectious diseases, the use of appropriate drug combinations presents several advantages, since this infection warrants superior microbicidal activity while reducing the risk of drug resistance emergence, given the very low likelihood of developing simultaneous resistance to two or more unrelated targets [7,8]. The current search for compounds characterized by a multi-target profile is based on this rationale, with the additional advantage of requiring lower effort, time, cost, and resources to optimize the absorption, distribution, metabolism excretion and toxicity ADME-T profile of a single multi-targeting new molecular entity (NME) compared to those required to identify multiple separate NMEs for combination therapy. The combination of a multi-target affinity profile in a single NME is therefore a challenging but widely accepted strategy to overcome rapid development of resistance and to increase the therapeutic lifespan of drugs in both anti-infective and anticancer chemotherapies [9,10,11].

For the rapid search and identification of novel therapeutic options, drug repurposing has emerged as a valuable approach in several fields [12,13,14], in particular for infectious diseases, including TB [15]. Here, we present our in silico screening approach for the identification of Food and Drug Administration (FDA)-approved drugs endowed with previously undetermined antimycobacterial activity and with potential multi-targeting profiles. We previously discovered inhibitors of the zinc-dependent metalloprotease-1 (Zmp1), a virulence factor essential for *Mtb* survival inside macrophages, which were proven to be able to impair the survival of *Mtb* inside macrophages with no activity on axenic *Mtb* [16]. In the search of multitargeting compounds, we aimed to discover compounds able to both kill *Mtb* inside macrophages and under axenic conditions. Based on this rationale, we identified a second enzyme, peptide deformylase (PDF), that was chosen for our virtual screening campaign based on its role in *Mtb* growth and its possible active-site similarities with Zmp1 (both are metalloenzymes) [17,18]. The FDA-approved drugs were screened in silico against PDF and Zmp1. The drugs predicted to inhibit both enzymes were subjected to a phenotypical investigation of their antitubercular potential as a direct effect in axenic culture and during infection of peripheral blood mononuclear cells (PBMCs), with granuloma-like structure (GLS) as a formation control. From our screening campaign, several FDA-approved drugs showed interesting antimycobacterial activity worth further investigation with the goal of enriching the therapeutic armamentarium for the treatment of TB.

## 2. Results

### 2.1. In Silico Screening and Antimycobacterial Activity of the Selected Compounds under Axenic Conditions

**Figure 1:**
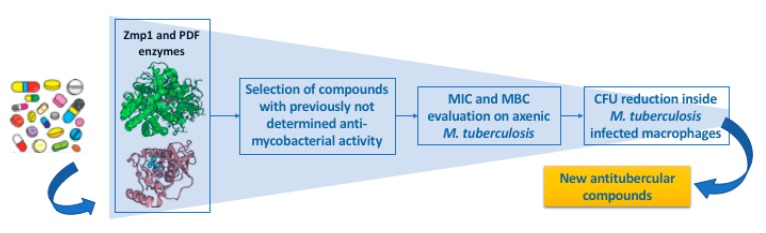
Work-flow of the in silico structure-based/phenotypic screening campaign of FDA-approved drugs. Zmp1: Zinc-dependent metalloprotease-1, PDF: peptide deformylase, MIC: minimum inhibitory concentration, MBC: minimum bactericidal concentration, CFU: colony forming unit.

The screening campaign of the FDA-approved drugs was performed as illustrated in the workflow presented in Figure 1. This integrated screening was designed by combining in silico and in vitro experiments in order to identify drugs possessing antimycobacterial activity. In the first step of the screening, we performed an accurate in silico analysis taking into account two enzymatic targets: (i) The virulence factor Zmp1, a zinc-protease essential for *Mtb* survival inside macrophages, since it interferes with the phagosome maturation by inhibiting the inflammasome [16,19,20,21], and (ii) the PDF enzyme, a ubiquitous bacterial iron-containing enzyme, responsible for the cleavage of the formyl group from nascent polypeptides [22,23]. Interestingly, these two metalloenzymes share a similar arrangement of amino acidic composition of their active sites. In particular, two His residues are involved in metal coordination, while the third residue completing the metal coordination is Glu for Zmp1 and Cys for PDF. Moreover, Zmp1 has no human counterpart and PDF presents a different catalytic site with respect to the human counterpart (PDF, mitochondrial) and other human related metalloenzymes.

Hence, PDF and Zmp1, along with a library of FDA-approved drugs, were employed in our high-throughput docking (HTD) campaign. Compounds showing either a docking score for both enzymes (<−8.00 kcal/mol coupled with a satisfactory ΔG_bind_) or very high score for at least one enzyme were selected for phenotypic screening (see experimental section for further details). The list of compounds showing appropriate scores is reported in Supplementary Materials (Table S1). We identified 73 compounds that matched our filters. Among them, compounds with previously reported antitubercular activity were not submitted to phenotypic screening.

**Figure 2:**
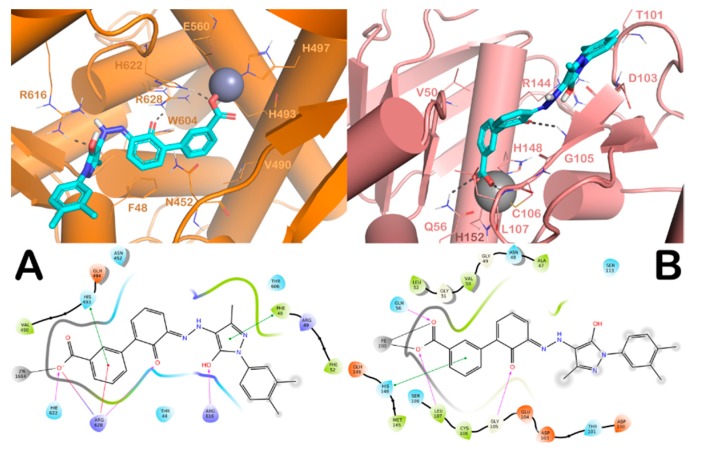
(**A**) Docking results for the representative compound eltrombopag. Putative binding modes of eltrombopag into Zmp1 and (**B**) PDF. The key residues of the binding sites are represented by lines and the metal ions are represented by spheres for Zn^2+^ and Fe^2+^, respectively. The H-bonds are depicted as dotted lines and the metal coordination bonds are represented by sticks. Pictures were prepared by PyMOL and Ligand Interaction Diagram implemented in Maestro.

The potential binding modes of one representative drug, eltrombopag, into the active site of both selected enzymes is shown in Figure 2. In both enzymes, the carboxylic group of eltrombopag coordinates the metal ion, while the aromatic carboxylic moiety is engaged in stacking interactions (double stacking with R628 (cation–π) and H493 (π–π) for Zmp1 and π–π stacking with H148 for PDF). These interactions were reported to be important for inhibiting the enzymes by known inhibitors [16,18]. Further contacts were found to stabilize the proposed binding mode in both enzymes comprising H-bonds and π–π stacking, as depicted in Figure 2A,B. In particular, the carbonyl in the central region of eltrombopag forms an H-bond with R628, while the terminal moiety establishes π–π stacking with F48 and an H-bond with R616 in the Zmp1 active site. In the PDF binding site, eltrombopag forms π–π stacking with H148 and two H-bonds with Q56 and the backbone of L107; its central region establishes a further H-bond with the backbone of G105. In the following steps of the protocol, drugs satisfying computational filtering criteria (73 FDA-approved drugs) were subjected to a second filter in order to eliminate all compounds with previously assessed antimycobacterial activity (Table S1). From the in silico screening campaign, 29 compounds were submitted for phenotypic antimycobacterial evaluation. 

The antimycobacterial activity of the selected compounds was investigated using *Mtb* H37Rv and other two *Mtb* clinical isolates (H3 and Beijing) belonging to different phylogeographic lineages [24,25]. The minimum inhibitory concentration (MIC) and minimum bactericidal concentration (MBC) in axenic culture were determined and these results are reported in Table 1. Of the compounds tested, 20 showed at least moderate activity (MIC = 100 μM), with 5 compounds showing MIC values of < 12.5 μM.

### 2.2. Treatment of Mtb-Infected PBMCs with Selected Drugs

**Figure 3:**
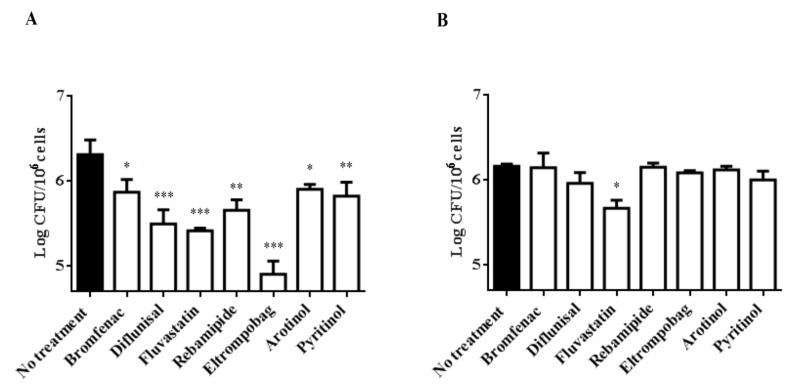
(**A**) Colony forming units (CFUs) after treatment of infected peripheral blood mononuclear cells PBMCs with different compounds. The efficacy of the selected drugs during infection was evaluated by calculating the colony forming units (CFUs) after 10 days of infection of the PBMCs with *M. tuberculosis* H37Rv at multiplicity of infection (MOI) 1:1. All compounds were added to the culture 3 days post-infection for one week (bromfenac 200 µM, diflunisal 100 µM, fluvastatin 50 µM, rebamipide 200 µM, eltrompobag 12.5 µM, arotinolol 6.25 µM, and pyritinol 100 µM). As a negative control, non-treated cells were used (no treatment). The CFU results showed a significant decrease in CFUs after applied treatment. *** *p* < 0.0001 for fluvastatin, eltrompobag, and diflunisal vs. control (no treatment), ** *p* < 0.01 for pyritinol and rebamipide vs. control, * *p* < 0.1 for bromfenac and arotinolol vs. control. (**B**) CFUs after treatment of infected PBMCs with different compounds at concentration (0.5 × MIC). The possible efficacy of these compounds on the host cells beyond the direct effect on the Mycobacteria was evaluated by adding the compounds to PBMCs 3 days post-infection with *M. tuberculosis* H37Rv with MOI 1:1 at specific concentrations (bromfenac 50 µM, fluvastatin 12.5 µM, pyritinol 25 µM, rebamipide 50 µM, eltrompobag 3.125 µM, arotinolol 1.56 µM, and diflunisal 25 µM) corresponding to 0.5 × MIC. Then, the CFUs were evaluated 7 days post-treatment. The obtained results showed no difference in CFUs between the treated and non-treated PBMCs, except for fluvastatin, which demonstrated a significant decrease in CFUs with respect to the non-treated PBMCs (* *p* < 0.1).

To evaluate the antitubercular activity of the most interesting drugs identified in the previous steps, including those showing good docking scores only against Zmp1 (bromfenac and rebamipide), we implemented an in vitro model of infection, where PBMCs isolated from donors were infected with *Mtb* and the activity was measured as a reduction in colony forming units (CFUs) compared to untreated *Mtb*-infected cells. As shown in Figure 3A, bromfenac, diflunisal, fluvastatin, rebamipide, eltrompobag, arotinolol, and pyritinol significantly restricted *Mtb* growth, as shown by the reduction in total CFUs measured after seven days of treatment, at concentrations twice the MIC (2 × MIC) for each drug. The CFU results showed that eltrompobag and fluvastatin were the most potent drugs regarding CFU reduction (1.4 and 1.0 log CFU reduction, respectively) with eltrombopag able to reduce CFUs by 22.5% with respect to the control (non-treated *Mtb*-infected PBMC). The other compounds tested were able to reduce CFUs to a lesser, yet significant, extent, ranging between 0.4 and 0.9 log CFU. To determine whether these compounds were able to exert activity on host cells, thereby triggering host-dependent antimicrobial activity, *Mtb*-infected PBMCs were treated with the selected compounds at a reduced concentration (0.5 × MIC) (Figure 3B). The results obtained after seven days of treatment showed that none of the compounds were able to restrict the *Mtb* intracellular growth at 0.5 × MIC concentration, with the only notable exception being Fluvastatin, which induced a significant reduction in CFUs.

### 2.3. Granuloma-Like Structure (GLS) Formation before and after Treatment of Infected PBMCs

**Figure 4:**
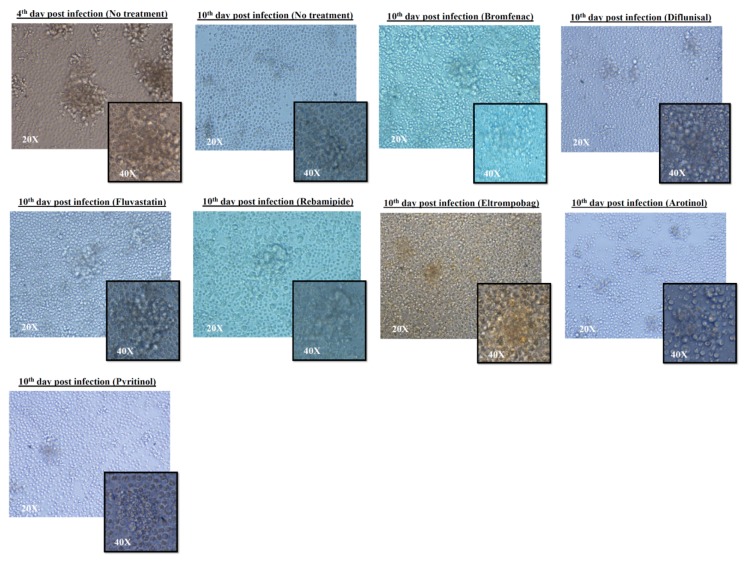
Inverted microscope images of the *M. tuberculosis* H37Rv-infected PBMCs during treatment. The images in the figure show the formation of granuloma-like structures (GLSs) of the infected PBMCs pre- and post-treatment with different selected compounds (bromfenac 200 µM, diflunisal 100 µM, fluvastatin 50 µM, rebamipide 200 µM, eltrompobag 12.5 µM, arotinolol 6.25 µM, and pyritinol 100 µM). The images were acquired at 20× and 40× magnification. The microscopic images captured from different angles show no differences in the GLSs formed pre- and post-treatment in terms of volume and number.

Infection of PBMC with *Mtb* led to formation of GLSs, which are agglomerates of polymorphonucleates and lymphocytes around *Mtb*-infected macrophages [26]. These GLSs can be observed by light microscopy daily and provide relevant information regarding the ability of the system to restrict *Mtb* replication [27]. Non-major differences were observed in the size, volume, number, and features of the GLSs treated with the selected compounds (bromfenac, diflunisal, fluvastatin, rebamipide, eltrompobag, arotinolol, and pyritinol) (Figure 4).

## 3. Discussion

Drug repurposing or drug repositioning (the latter referring to a drug approved for one disease which is used as a structural template for the synthesis of derivatives active against another disease), coupled with phenotypic screening on whole cell, are powerful approaches used for the discovery of new anti-infective agents. Drug repurposing drastically reduces the time and effort necessary for the approval a new drug, while direct testing on phenotypic cells allows for the selection and prioritization of specific compounds to reach their targets inside the cell in further studies. This is a critical issue for the identification of novel therapeutic approaches against *Mtb* due to the impervious nature of its membrane. Through a virtual screening approach, in which FDA-approved drugs were screened on the basis of their potential affinity against Zmp1 and PDF, two metalloenzymes important for the virulence and survival of *M. tuberculosis*, we discovered a series of marketed drugs with interesting antitubercular activity, not only against the *Mtb* reference strain, but also against other clinically isolated strains. The *Mtb* H3 strain belongs to the phylogeographic lineage 4 (Euro-American lineage) and the *Mtb* Beijing strain belongs to phylogeographic lineage 2 (East Asian lineage), with the latter often associated with a drug-resistant phenotype [25,28]. The reference strain and the two clinical isolates presented significant differences in terms of pathogenic potential and biological features, thus explaining the differences in the MIC results obtained for several drugs. Eltrombopag, an orally active thrombopoietin receptor agonist with megakaryopoiesis-stimulating activity, and arotinolol, a medication in the class of mixed α/β blockers used in the treatment of high blood pressure and essential tremor were the most potent compounds of the series identified in this study against *Mtb* in macrophages and axenic culture, respectively. These compounds could be the subjects of a drug repositioning campaign in order to further improve their potency and to eliminate off-target liability. Their potential multi-targeting activity profiles would provide a rational basis for the optimization and development of antitubercular agents, with low liability to select drug-resistant *Mtb* strains. 

Our screening campaign also uncovered the antimycobacterial activity of other drugs, namely bromfenac, diflunisal, and fluvastatin, with different multi-targeting activity.

Rebamipide, an amino acid analogue 2-(1*H*)-quinolinone known for its gastroprotective activity, pyritinol, a Vit B6 analog, and sofalcone, a mucosal protective agent, showed moderate anti-TB activity. On the other hand, carfilzomib, although being highly active in the phenotypic assay, is an inhibitor of human proteasomes with high inherent toxicity [29].

Diflunisal (a salicylic acid derivative) and bromfenac, although both possessing analgesic and anti-inflammatory activity and belonging to the class of non-steroidal anti-inflammatory drugs (NSAIDs), showed different antitubercular activity levels; diflunisal was more potent, not only in axenic culture, but also during infection in PBMCs and was able to reduce CFUs by 0.9 log, which was a greater level of activity than bromfenac (0.5 log CFU reduction). 

Fluvastatin, as reported in this study, and atorvastatin, are members of the statin drug class and are used to treat hypercholesterolemia and prevent cardiovascular diseases. Its mechanism of action involves blocking the liver enzyme hydroxy-methyl-glutaryl-CoA (HMG-CoA) reductase, which facilitates an important step in cholesterol synthesis. The data presented here are extremely relevant, since both fluvastatin and diflunisal could be considered antitubercular candidates endowed with host-directed activity [30], and our studies using 0.5 MIC concentration confirmed that fluvastatin could target not only the pathogen but also the host cell, and increase the ability of infected cells to eliminate *Mtb*. In fact, previous studies demonstrated that cholesterol inhibition by statins within phagosomal membranes could promote host-induced autophagy, thereby improving host protection against TB [31].

A reduction in PGE2 is considered one mechanistic explanation underlying the anti-inflammatory benefit of NSAIDs in the context of *M. tuberculosis* infection [31]. Moreover, certain NSAIDs demonstrated inhibitory properties toward actively replicating, dormant, and drug-resistant clinical isolates of *Mtb* cells, as PGE2 inhibits phagocytosis and bacterial killing at a late stage of *Mtb* infection.

In conclusion, we presented an integrated screening protocol combining in silico and in vitro approaches to uncover the antimycobacterial potential of existing drugs. Our screening protocol provided a high number of active compounds (69%) against *Mtb*, with a series of marketed drugs (eltrombopag, arotinolol, diflunisal, bromfenac, and fluvastatin) possessing significant antitubercular activity levels, as assessed by different in vitro tests. Accordingly, these findings open the door for the exploration of the therapeutic potential of some FDA-approved drugs to fight TB. 

## 4. Materials and Methods

### 4.1. In Silico Screening

#### 4.1.1. Protein Preparation

The crystal structures of the virulence factor zinc-dependent metalloprotease-1 (Zmp1) (PDB-ID: 3ZUK, resolution of 2.6 Å; the protein was crystallized with the inhibitor phosphoramidon) [19] and the peptide deformylase (PDF) (PDB-ID: 3E3U, resolution of 1.56 Å.; the protein was crystallized with inhibitor **16a**) [18] were downloaded from Protein Data Bank and prepared by means of Protein Preparation Wizard implemented in Maestro Suite [32], as previously reported [16,33]. The materials used for the crystallization process were removed, keeping the inhibitors present in both selected metalloenzymes. Before the optimization protocol regarding the PDF enzyme, we removed the Ni^2+^ ion, replacing it with an Fe^2+^ ion to resemble the wild-type enzyme.

#### 4.1.2. Database Preparation

The FDA-approved drug dataset was taken from the ZINC database (http://zinc15.docking.org/) (~3000 drugs) and prepared by means of Macromodel [34] and LigPrep [35], as described by us [33,36,37]. In particular, all of the drugs were minimized using MacroModel by employing the force field OPLSAA_2005 [38]. The generalized born/surface area (GB/SA) solvation model for simulating the solvent effect was used with ‘‘no cutoff’’ for non-bonded interactions. The Polak-Ribiere conjugate gradient (PRCG) method (5000 maximum iterations and 0.001 gradient convergence threshold) was employed. Compounds were then submitted to the LigPrep program, generating possible ionization states at pH 7.4 ± 0.2.

#### 4.1.3. High-Throughput Docking (HTD) Details

Grid-Based Ligand Docking with Energetics (Glide) was employed for the high-throughput docking (HTD) procedure using the FDA-approved drug database and the proteins prepared as mentioned above by applying Glide extra precision (XP) method [39]. Energy grids were prepared using default value of protein atom scaling factor (1.0 Å) within a cubic box centered on crystallized inhibitors. After that, the FDA-approved drug database was docked into the enzymes with default parameters considering the metal constraint options to generate the possible geometries for the metal-coordination bonds. The number of poses entered into the post-docking minimization was set to 50. The Glide XP score was evaluated for both proteins. The interactions of the drugs with both proteins were assessed by using a ligand-interaction diagram available from the Maestro suite and a script for displaying hydrophobic interactions (display_hydrophobic_interactions.py) downloaded from Schrödinger website and implemented in Maestro. Furthermore, the HTD protocol was implemented with the calculation of ligand-binding energy performed by Prime software [40], as previously described [41,42,43]. In order to obtain further accuracy for our protocol, the calculation of relative ligand-binding energy (ΔG_bind_) between a ligand and its receptor offered a worthwhile post-scoring approach for prioritizing the screened hits with a lower ΔG_bind_. The molecular mechanical (MM)-GBSA approach combined the MM energies with a continuum solvent generalized born (GB) model for polar solvation, as well as a solvent-accessible surface area (SASA) for non-polar solvation. Accordingly, the representative docked poses obtained through the molecular docking studies were submitted to Prime protocol to obtain the final ranking of the drugs against the selected enzymes. The final selection was performed by combining visual inspection (interaction of drugs with key residues in the binding sites of both selected enzymes and metal coordination bonds), docking scores (for both enzymes, we selected compounds with scores of <−8.00 kcal/mol; this value was chosen considering the representative inhibitors of both enzymes) coupled with a satisfactory ΔG_bind_ (<−40 kcal/mol, that is, the relative ligand-binding energy as calculated by Prime MM-GBSA). Moreover, we only purchased drugs with no previously determined antimycobacterial activity (compounds labeled as not previously determined in Table S1). Moreover, in order to validate the docking protocol, we redocked phosphoramidon (crystallized ligand for Zmp1) and compound **16a** (crystallized ligand for PDF [18]). We found that the docking protocol correctly accommodated the mentioned ligands (data not shown). Moreover, due to the absence of a sufficient number of potent inhibitors (the most potent was developed by us [16]), it was not possible to provide a calculation for the enrichment factor of the selected docking protocol for Zmp1, since the results could be not statistically relevant. Regarding the enrichment factor for PDF, the most potent inhibitors were derived from the paper of the crystal structure (14 compounds) [18], so the docking protocol was able to distinguish between the active molecules. Validation using the decoy set generated from these 14 compounds was not performed, since it would not provide statistically relevant results. 

### 4.2. Measurement of the Minimum Inhibitory Concentration (MIC) and Minimum Bactericidal Concentration (MBC) of All Drugs

The antimycobacterial activity evaluation of all of the drugs against the *Mtb* strains (H37Rv, H3, Beijing, China) was carried out in Middelbrook 7H9 broth medium (Difco Becton-Dickinson, Franklin Lakes, NJ, USA), supplemented with 0.2% glycerol (Sigma-Aldrich, St. Louis, MO, USA), 10% Albumin-Dextrose-Catalase (ADC) (Becton-Dickinson, Franklin Lakes, NJ, USA), and 0.05% Tween 80 (Sigma-Aldrich, St. Louis, MO, USA) at 37 °C [44,45,46]. A series of decreasing concentrations of the selected compounds (100, 50, 25, 12.5, 6.25, 3.125, 1.56, and 0 µM) was prepared and inoculated with ≈5 × 10^3^ CFU/mL. After 14 days of incubation, the MIC was determined by microplate alamar blue assay [47]. An aliquot of the culture was collected to determine the CFUs at the MIC and higher concentrations to calculate the MBC for each compound. The aliquot was seeded on solid media (7H11) supplemented with 10% Oleic Acid-Albumin-Dextrose-Catalase (OADC) (Microbiol, Uta, Italy) after 14 days of incubation at 37 °C.

### 4.3. PBMCs Isolation from a Human Blood

Human blood was collected from healthy volunteers after confirmed written consent stating that the blood donor was not subject to any therapy or taking any other drugs. Peripheral blood mononuclear cells (PBMCs) were isolated from the whole blood by Ficoll human lymphocyte^®^ (CEDARLANE, Ontario, Canada) density gradient cell separation media using standard protocol [26,48]. The extracted PBMCs were suspended with Roswell Park Memorial Institute (RPMI) 1640 medium (Euroclone, Milan, Italy) enriched with 10% fetal bovine serum (FBS) and 2 mM glutamine (Euroclone, Milan, Italy) without antibiotics. Finally, the extracted PBMCs were plated onto 48-well culture plates treated with polystyrene (Nest Biotech, Wuxi, China) at a final concentration of 1.2 × 10^6^ cell/mL.

### 4.4. Infection of the PBMCs Extracted Cells

The isolated PBMCs were infected with the *Mtb* H37Rv reference strain at multiplicity of infection (MOI) 1:1, which normalized depending on the percentage of the monocytes from the total PBMCs [49]. Three days post-infection, the compounds were added to the supernatant of the infected cells at different concentrations depending on the minimum inhibitory concentration previously observed and considering the cytotoxicity (CC_50_) for each compound to avoid any toxic adverse effects. First, the compounds were used at 2 × MIC concentrations (bromfenac 200 µM, diflunisal 100 µM, fluvastatin 50 µM, rebamipide 200 µM, eltrompobag 12.5 µM, arotinolol 6.25 µM, and pyritinol 100 µM) or by repeating the infection as previously described at concentration 0.5 × MIC in the next step to evaluate the possible host-directed effects of these compounds (bromfenac 50 µM, diflunisal 25 µM, fluvastatin 12.5 µM, rebamipide 50 µM, eltrompobag 3.125 µM, arotinolol 1.56 µM, and pyritinol 25 µM). Finally, the colony forming units (CFUs) were calculated for all compounds after 7 days of treatment. Briefly, PMBCs were lysed in 0.01% triton X-100, serial dilutions prepared in PBS/0.05% Tween80, and aliquots were plated on 7H11/OADC/agar medium [50]. Results were expressed as LogCFU/106 cells.

### 4.5. Image Acquisition for Granuloma-Like Structure (GLS)

Formation of the granuloma-like structures (GLSs) was induced after PBMC infection, as previously described with the *Mtb* H37Rv reference strain [27,50]. The images of the aggregates were acquired using an inverted microscope (Nikon Eclipse TS 100, Melville, NY, USA) with Apodized Dark Low (ADL) 20× and 40× objective lens at 3 days post-infection and 7 days after treatment with all of the selected compounds (bromfenac 200 µM, diflunisal 100 µM, fluvastatin 50 µM, rebamipide 200 µM, eltrompobag 12.5 µM, arotinolol 6.25 µM, and pyritinol 100 µM). Finally, the number and volume of GLSs formed were analyzed by observing different microscopic spaces for each condition. 

### 4.6. Statistics

All experiments were replicated at least three times. Microsoft Excel (2010) and Graphpad Prism software version 6 (GraphPad prism 6 software, GraphPad Software Inc, CA, USA) were used to analyze the data. All data were expressed as the mean plus SD and analyzed by one-way or two-way ANOVA comparison tests followed by the appropriate correction, as specified in the caption under each figure.

## Figures and Tables

**Table 1:** The minimum inhibitory concentration (MIC) and the minimum bactericidal concentration (MBC) of the selected compounds against *M. tuberculosis* H37Rv reference strains and two other clinical isolated strains (H3 and Beijing). After the computed screening, the three different *M. tuberculosis* strains were grown in a liquid medium at an initial concentration of ≈ 5 × 10^3^ CFU/mL in contact with a decreasing concentration of selected compounds (100, 50, 25, 12.5, 6.25, 3.125, 1.56, and 0 µM). Different results were obtained after 14 days of incubation at 37 °C for each strain.

Drug	MIC μM (H37Rv)	MBC μM (H37Rv)	MIC μM (H3)	MBC μM (H3)	MIC μM (Beijing)	MBC μM (Beijing)
Bromfenac	100	>100	100	>100	50	100
Naratriptan	>100	n.d.	>100	n.d.	>100	n.d.
Sofalcone	100	>100	50	100	50	100
Fosinopril	50	100	>100	n.d.	100	>100
Carfilzomib	1.56	3.125	*	n.d.	*	n.d.
Pemetrexed	25	50	>100	n.d.	>100	n.d.
Sitagliptin	>100	n.d.	>100	n.d.	>100	n.d.
Tamsulosin	>100	n.d.	>100	n.d.	>100	n.d.
Nelarabine	50	100	50	100	>100	n.d.
Pazopanib	>100	n.d.	>100	n.d.	>100	n.d.
Fluvastatin	25	50	50	100	25	50
Pyritinol	50	100	>100	n.d.	50	100
Ledipasvir	>100	n.d.	12.5	50	>100	n.d.
Cellcept	12.5	25	50	100	50	100
Biotin	>100	n.d.	>100	n.d.	>100	n.d.
Picosulfuric acid	25	50	>100	n.d.	>100	n.d.
Peramivir	100	>100	>100	n.d.	>100	n.d.
Rebamipide	100	>100	>100	n.d.	100	>100
Pitavastatin	50	100	>100	n.d.	100	>100
Eltrombopag	6.25	12.5	50	100	12.5	25
Nintedanib	25	50	25	50	12.5	25
Mitiglinide	25	50	>100	n.d.	50	100
Bicalutamide	>100	n.d.	>100	n.d.	>100	n.d.
Arotinolol	3.125	6.25	50	100	25	50
Diflunisal	50	100	50	100	25	50
Amifostine	50	100	100	>100	>100	n.d.
Fosamax	12.5	25	>100	n.d.	>100	n.d.
Cidofovir	>100	n.d.	>100	n.d.	>100	n.d.
Ranelic acid	>100	n.d.	>100	n.d.	>100	n.d.

1.25–12.5 μM25–50 μM100 μM>100 μMn.d.

* Carfilzomib was not tested on H3 and Beijing strains due to poor aqueous stability; n.d. = not determined.

## Supplementary Materials

The following are available online, Table S1: Full list of FDA-approved compounds selected from virtual screening.

## References

[1] World Health Organization (2019). Global Tuberculosis Report 2018.

[2] Dobbs T.E., Webb R.M. (2017). Chemotherapy of Tuberculosis. Microbiol. Spectr..

[3] Dheda K., Gumbo T., Maartens G., Dooley K.E., Murray M., Furin J., Nardell E.A., Warren R.M. (2019). Lancet Respiratory Medicine drug-resistant tuberculosis Commission, g., The Lancet Respiratory Medicine Commission: 2019 update: Epidemiology, pathogenesis, transmission, diagnosis, and management of multidrug-resistant and incurable tuberculosis. Lancet Respir. Med..

[4] Dheda K., Gumbo T., Maartens G., Dooley K.E., McNerney R., Murray M., Furin J., Nardell E.A., London L., Lessem E. (2017). The epidemiology, pathogenesis, transmission, diagnosis, and management of multidrug-resistant, extensively drug-resistant, and incurable tuberculosis. Lancet Respir. Med..

[5] Siddiqi S., Takhar P., Baldeviano C., Glover W., Zhang Y. (2007). Isoniazid induces its own resistance in nonreplicating Mycobacterium tuberculosis. Antimicrob. Agents Chemother..

[6] Raghunandanan S., Jose L., Kumar R.A. (2018). Dormant Mycobacterium tuberculosis converts isoniazid to the active drug in a Wayne’s model of dormancy. J. Antibiot. (Tokyo).

[7] Smith T., Wolff K.A., Nguyen L. (2013). Molecular biology of drug resistance in Mycobacterium tuberculosis. Curr. Top. Microbiol. Immunol..

[8] Torfs E., Piller T., Cos P., Cappoen D. (2019). Opportunities for Overcoming Mycobacterium tuberculosis Drug Resistance: Emerging Mycobacterial Targets and Host-Directed Therapy. Int. J. Mol. Sci..

[9] Oldfield E., Feng X. (2014). Resistance-resistant antibiotics. Trends Pharmacol. Sci..

[10] De Oliveira Viana J., Ishiki H.M., Scotti M.T., Scotti L. (2018). Multi-Target Antitubercular Drugs. Curr. Top. Med. Chem..

[11] Li K., Schurig-Briccio L.A., Feng X., Upadhyay A., Pujari V., Lechartier B., Fontes F.L., Yang H., Rao G., Zhu W. (2014). Multitarget drug discovery for tuberculosis and other infectious diseases. J. Med. Chem..

[12] Fong W., To K.K.W. (2019). Drug repurposing to overcome resistance to various therapies for colorectal cancer. Cell. Mol. Life Sci..

[13] Rommer P.S., Sellner J. (2019). Repurposing multiple sclerosis drugs: A review of studies in neurological and psychiatric conditions. Drug Discov. Today.

[14] Cha Y., Erez T., Reynolds I.J., Kumar D., Ross J., Koytiger G., Kusko R., Zeskind B., Risso S., Kagan E. (2018). Drug repurposing from the perspective of pharmaceutical companies. Br. J. Pharmacol..

[15] Zheng W., Sun W., Simeonov A. (2018). Drug repurposing screens and synergistic drug-combinations for infectious diseases. Br. J. Pharmacol..

[16] Paolino M., Brindisi M., Vallone A., Butini S., Campiani G., Nannicini C., Giuliani G., Anzini M., Lamponi S., Giorgi G. (2018). Development of Potent Inhibitors of the Mycobacterium tuberculosis Virulence Factor Zmp1 and Evaluation of Their Effect on Mycobacterial Survival inside Macrophages. ChemMedChem.

[17] Teo J.W., Thayalan P., Beer D., Yap A.S., Nanjundappa M., Ngew X., Duraiswamy J., Liung S., Dartois V., Schreiber M. (2006). Peptide deformylase inhibitors as potent antimycobacterial agents. Antimicrob. Agents Chemother..

[18] Pichota A., Duraiswamy J., Yin Z., Keller T.H., Alam J., Liung S., Lee G., Ding M., Wang G., Chan W.L. (2008). Peptide deformylase inhibitors of Mycobacterium tuberculosis: Synthesis, structural investigations, and biological results. Bioorg. Med. Chem. Lett..

[19] Ferraris D.M., Sbardella D., Petrera A., Marini S., Amstutz B., Coletta M., Sander P., Rizzi M. (2011). Crystal structure of Mycobacterium tuberculosis zinc-dependent metalloprotease-1 (Zmp1), a metalloprotease involved in pathogenicity. J. Biol. Chem..

[20] Lazarevic V., Martinon F. (2008). Linking inflammasome activation and phagosome maturation. Cell. Host Microbe.

[21] Master S.S., Rampini S.K., Davis A.S., Keller C., Ehlers S., Springer B., Timmins G.S., Sander P., Deretic V. (2008). Mycobacterium tuberculosis prevents inflammasome activation. Cell Host Microbe.

[22] Sharma A., Khuller G.K., Sharma S. (2009). Peptide Deformylase—A promising therapeutic target for tuberculosis and antibacterial drug discovery. Expert Opin. Ther. Targets.

[23] Sharma A., Khuller G.K., Kanwar A.J., Sharma S. (2010). Therapeutic potential of peptide deformylase inhibitors against experimental tuberculosis. J. Infect..

[24] Barbier M., Wirth T. (2016). The Evolutionary History, Demography, and Spread of the Mycobacterium tuberculosis Complex. Microbiol. Spectr..

[25] Romagnoli A., Petruccioli E., Palucci I., Camassa S., Carata E., Petrone L., Mariano S., Sali M., Dini L., Girardi E. (2018). Clinical isolates of the modern Mycobacterium tuberculosis lineage 4 evade host defense in human macrophages through eluding IL-1β-induced autophagy. Cell Death Dis..

[26] Bhavanam S., Rayat G.R., Keelan M., Kunimoto D., Drews S.J. (2018). Characterization of immune responses of human PBMCs infected with Mycobacterium tuberculosis H37Ra: Impact of donor declared BCG vaccination history on immune responses and M. tuberculosis growth. PLoS ONE.

[27] Je S., Quan H., Na Y., Cho S.N., Kim B.J., Seok S.H. (2016). An in vitro model of granuloma-like cell aggregates substantiates early host immune responses against Mycobacterium massiliense infection. Biol. Open.

[28] Cox H.S., Kubica T., Doshetov D., Kebede Y., Rusch-Gerdess S., Niemann S. (2005). The Beijing genotype and drug resistant tuberculosis in the Aral Sea region of Central Asia. Respir. Res..

[29] Bard J.A.M., Goodall E.A., Greene E.R., Jonsson E., Dong K.C., Martin A. (2018). Structure and Function of the 26S Proteasome. Annu. Rev. Biochem..

[30] Palucci I., Delogu G. (2018). Host Directed Therapies for Tuberculosis: Futures Strategies for an Ancient Disease. Chemotherapy.

[31] Parihar S.P., Guler R., Khutlang R., Lang D.M., Hurdayal R., Mhlanga M.M., Suzuki H., Marais A.D., Brombacher F. (2014). Statin therapy reduces the mycobacterium tuberculosis burden in human macrophages and in mice by enhancing autophagy and phagosome maturation. J. Infect. Dis..

[32] Schrödinger (2015). Maestro.

[33] Zaccagnini L., Brogi S., Brindisi M., Gemma S., Chemi G., Legname G., Campiani G., Butini S. (2017). Identification of novel fluorescent probes preventing PrPSc replication in prion diseases. Eur. J. Med. Chem..

[34] Schrödinger (2015). MacroModel.

[35] Schrödinger (2015). LigPrep.

[36] Brindisi M., Brogi S., Giovani S., Gemma S., Lamponi S., De Luca F., Novellino E., Campiani G., Docquier J.D., Butini S. (2016). Targeting clinically-relevant metallo-beta-lactamases: From high-throughput docking to broad-spectrum inhibitors. J. Enzyme Inhib. Med. Chem..

[37] Brindisi M., Brogi S., Relitti N., Vallone A., Butini S., Gemma S., Novellino E., Colotti G., Angiulli G., Di Chiaro F. (2015). Structure-based discovery of the first non-covalent inhibitors of *Leishmania major* tryparedoxin peroxidase by high throughput docking. Sci. Rep..

[38] Jorgensen W.L., Maxwell D.S., TiradoRives J. (1996). Development and testing of the OPLS all-atom force field on conformational energetics and properties of organic liquids. J. Am. Chem. Soc..

[39] Friesner R.A., Banks J.L., Murphy R.B., Halgren T.A., Klicic J.J., Mainz D.T., Repasky M.P., Knoll E.H., Shelley M., Perry J.K. (2004). Glide: A new approach for rapid, accurate docking and scoring. 1. Method and assessment of docking accuracy. J. Med. Chem..

[40] Schrödinger (2015). Prime.

[41] Brindisi M., Gemma S., Kunjir S., Di Cerbo L., Brogi S., Parapini S., D’Alessandro S., Taramelli D., Habluetzel A., Tapanelli S. (2015). Synthetic spirocyclic endoperoxides: New antimalarial scaffolds. Medchemcomm.

[42] Brogi S., Fiorillo A., Chemi G., Butini S., Lalle M., Ilari A., Gemma S., Campiani G. (2017). Structural characterization of Giardia duodenalis thioredoxin reductase (gTrxR) and computational analysis of its interaction with NBDHEX. Eur. J. Med. Chem..

[43] D’Alessandro S., Alfano G., Di Cerbo L., Brogi S., Chemi G., Relitti N., Brindisi M., Lamponi S., Novellino E., Campiani G. (2019). Bridged bicyclic 2,3-dioxabicyclo[3.3.1]nonanes as antiplasmodial agents: Synthesis, structure-activity relationships and studies on their biomimetic reaction with Fe(II). Bioorg. Chem..

[44] Delogu G., Pusceddu C., Bua A., Fadda G., Brennan M.J., Zanetti S. (2004). Rv1818c-encoded PE_PGRS protein of Mycobacterium tuberculosis is surface exposed and influences bacterial cell structure. Mol. Microbiol..

[45] De Maio F., Maulucci G., Minerva M., Anoosheh S., Palucci I., Iantomasi R., Palmieri V., Camassa S., Sali M., Sanguinetti M. (2014). Impact of protein domains on PE_PGRS30 polar localization in Mycobacteria. PLoS ONE.

[46] De Maio F., Battah B., Palmieri V., Petrone L., Corrente F., Salustri A., Palucci I., Bellesi S., Papi M., Rubino S. (2018). PE_PGRS3 of Mycobacterium tuberculosis is specifically expressed at low phosphate concentration, and its arginine-rich C-terminal domain mediates adhesion and persistence in host tissues when expressed in Mycobacterium smegmatis. Cell Microbiol..

[47] Straniero V., Pallavicini M., Chiodini G., Zanotto C., Volonte L., Radaelli A., Bolchi C., Fumagalli L., Sanguinetti M., Menchinelli G. (2016). 3-(Benzodioxan-2-ylmethoxy)-2,6-difluorobenzamides bearing hydrophobic substituents at the 7-position of the benzodioxane nucleus potently inhibit methicillin-resistant Sa and Mtb cell division. Eur. J. Med. Chem..

[48] Kapoor N., Pawar S., Sirakova T.D., Deb C., Warren W.L., Kolattukudy P.E. (2013). Human granuloma in vitro model, for TB dormancy and resuscitation. PLoS ONE.

[49] Palucci I., Battah B., Salustri A., De Maio F., Petrone L., Ciccosanti F., Sali M., Bondet V., Duffy D., Fimia G.M. (2019). IP-10 contributes to the inhibition of mycobacterial growth in an ex vivo whole blood assay. Int. J. Med. Microbiol..

[50] Birkness K.A., Guarner J., Sable S.B., Tripp R.A., Kellar K.L., Bartlett J., Quinn F.D. (2007). An in vitro model of the leukocyte interactions associated with granuloma formation in Mycobacterium tuberculosis infection. Immunol. Cell Biol..

