# Supplementary material for: A Repurposing Approach for Uncovering the Anti-Tubercular Activity of FDA-Approved Drugs with Potential Multi-Targeting Profiles
Source: Molecules. 2019 Nov 29;24(23):4373. doi: 10.3390/molecules24234373 (PMC6930672; doi:10.3390/molecules24234373)
Supplement: Supplementary file 1 [file molecules-24-04373-s001.pdf]

molecules

# A Repurposing Approach for Uncovering the Anti-Tubercular Activity of FDA-Approved Drugs with Potential Multi-Targeting Profiles

**Basem Battah [1], Giulia Chemi [2], Stefania Butini [2], Giuseppe Campiani [2], Simone Brogi [3,]*, Giovanni Delogu [4,5] and Sandra Gemma [2,]***

[1] Fondazione Policlinico Universitario A. Gemelli, IRCCS Rome. Largo A. Gemelli, 0 00168 Rome, Italy; basem.battah.sc@hotmail.com
[2] Department of Biotechnology (DoE 2018-2022), Chemistry and Pharmacy (DoE 2018-2022), University of Siena, via Aldo Moro 2, 53100 Siena, Italy; giu.chemi@gmail.com (G.C.); stefania.butini@unisi.it (S.B.); campiani@unisi.it (G.C.)
[3] Department of Pharmacy, University of Pisa, via Bonanno 6, 56126 Pisa, Italy
[4] Institute of Microbiology, Università Cattolica del Sacro Cuore, Roma–Largo F. Vito, 1, 00168 Rome, Italy; giovanni.delogu@unicatt.it
[5] Mater Olbia Hospital, SS 125 Orientale Sarda, 07026 Olbia, Italy
* Correspondence: simone.brogi@unipi.it (S.B.); GEMMA@UNISI.IT (S.G.); Tel.: +39-050-2219613 (S.B.); +39-0577-234326 (S.G.)

# Supplementary Materials

**Table of contents**

Table S1 page S2

**Table S1.** Full list of FDA-compounds selected from virtual screening. The 29 compounds selected for the biological evaluation are underlined.

| **Compound** | **Structure** | **Test Performed Against *M. Tuberculosis*** | **Activity** |
|---|---|---|---|
| Acemetacin | 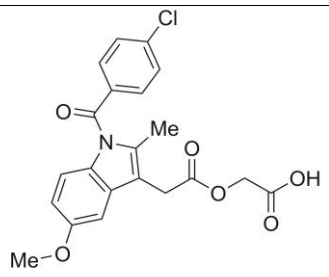 | HTS against *M. tuberculosis* in 7H9 media<br>HTS against H37Rv strain | inactive |
| Acenocoumarol | 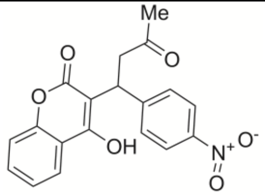 | HTS against H37Rv strain | active |
| Amifostine | 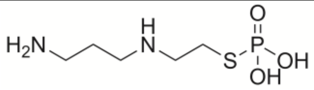 | no | not previously determined |
| Arotinolol | 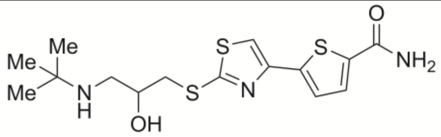 | no | not previously determined |
| Asacol | 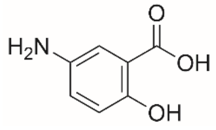 | HTS against H37Rv strain | inactive |
| Atovaquone | 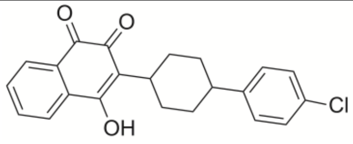 | HTS against H37Rv strain | active |
| Baclofen | 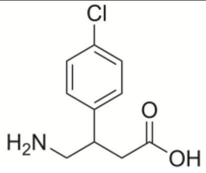 | HTS against H37Rv strain | inactive |
| Bezafibrat | 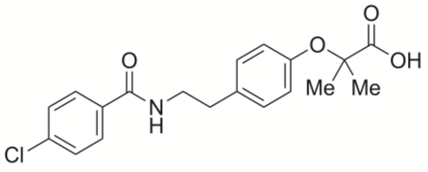 | HTS against *M. tuberculosis* in 7H9 media<br>HTS against H37Rv strain | inactive |

| | | | |
|---|---|---|---|
| Bicalutamide | 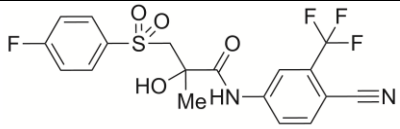 | no | not previously determined |
| Biotin | 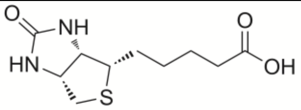 | no | not previously determined |
| Bromfenac* | 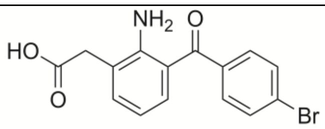 | no | not previously determined |
| Carfilzomib | 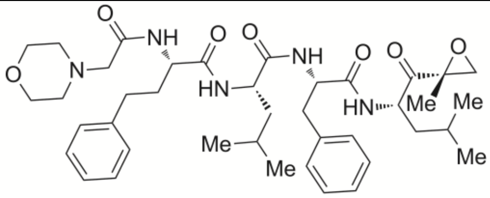 | no | not previously determined |
| Carzenide | 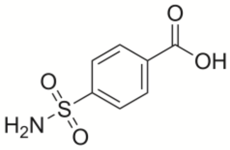 | HTS against *M. tuberculosis* in 7H9 media<br>HTS against H37Rv strain | inactive |
| Cellcept | 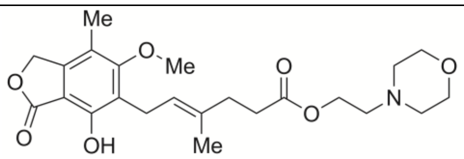 | no | not previously determined |
| Celecoxib | 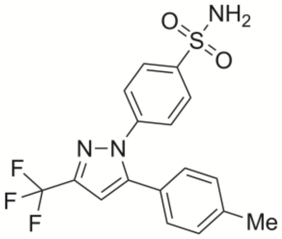 | HTS against *M. tuberculosis* in 7H9 media | inactive |
| Chloramphenicol | 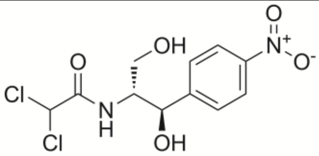 | HTS against H37Rv strain | active |
| Cidofovir | 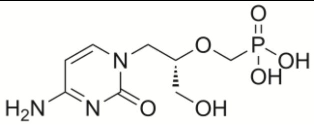 | no | not previously determined |
| Clonixin | 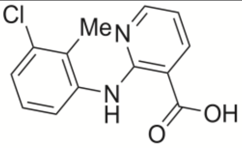 | HTS against *M. tuberculosis* in 7H9 media | inactive |

molecules MDPI

| | | | |
|---|---|---|---|
| Clorazepate | 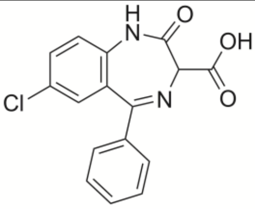 | no | not previously determined<br>not purchased |
| Diflunisal | 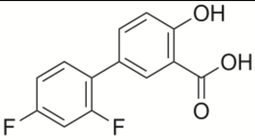 | no | not previously determined |
| Eltrombopag | 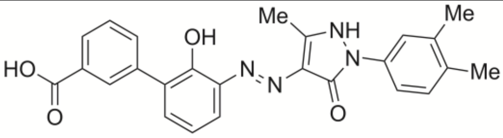 | no | not previously determined |
| Fenoldopam | 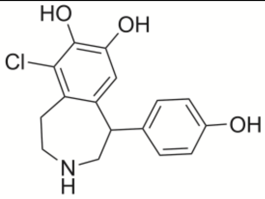 | HTS against H37Rv strain | inactive |
| Fenoterol | 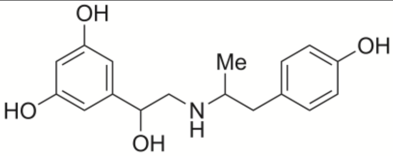 | HTS against *M. tuberculosis* in 7H9 media | inactive |
| Fentanyl | 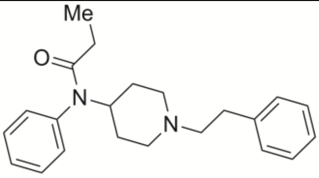 | no | not previously determined<br>not purchased |
| Florantyrone | 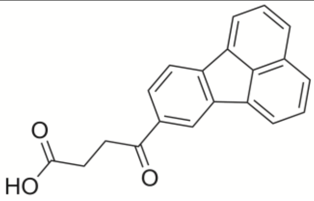 | no | not previously determined<br>not purchased |
| Flunarizine | 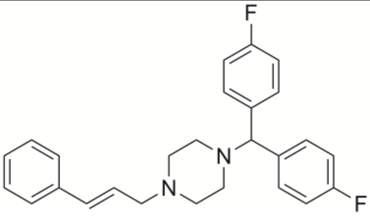 | HTS against H37Rv strain | active |
| Flurbiprofen | 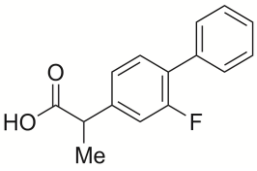 | HTS against *M. tuberculosis* in 7H9 media<br>HTS against H37Rv strain | inactive |

| | | | |
|---|---|---|---|
| Fluvastatin | 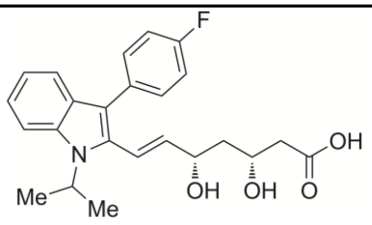 | no | not previously determined |
| Fosamax | 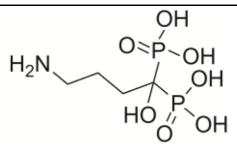 | no | not previously determined |
| Fosinopril | 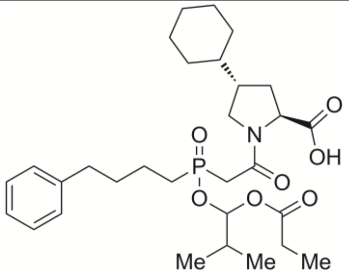 | no | not previously determined |
| Fosphenytoin | 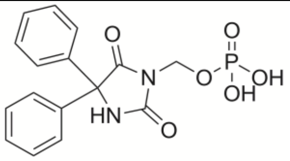 | no | not previously determined<br>not purchased |
| Furosemide | 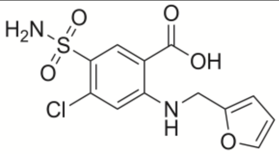 | HTS against H37Rv strain | active |
| Ibuprofen | 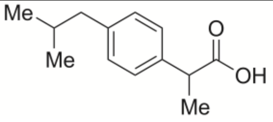 | HTS against H37Rv strain | inactive |
| Labetalol | 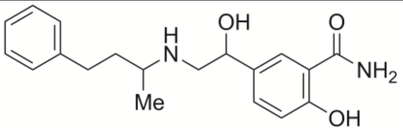 | HTS against H37Rv strain | inactive |
| Ketoprofen | 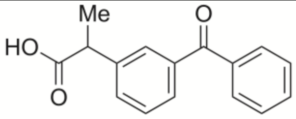 | HTS against H37Rv strain | inactive |
| Ketorolac | 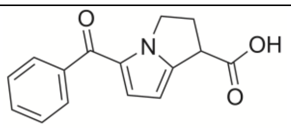 | HTS against H37Rv strain | inactive |

| | | | |
|---|---|---|---|
| Ledipasvir | 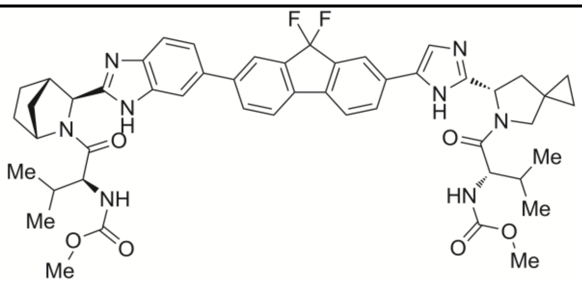 | no | not previously determined |
| Loperamide | 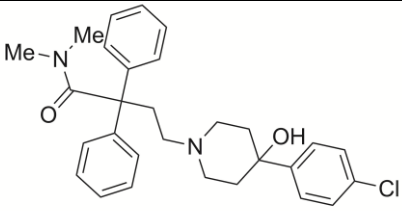 | HTS against H37Rv strain | active |
| Lopinavir | 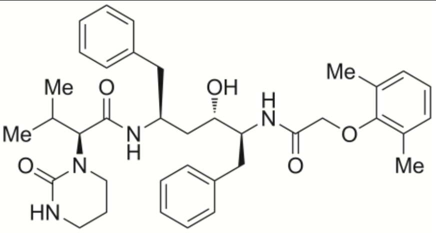 | no | not previously determined<br><br>not purchased |
| Mitiglinide | 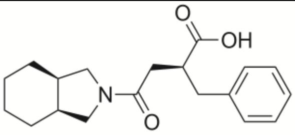 | no | not previously determined |
| Nadifloxacin | 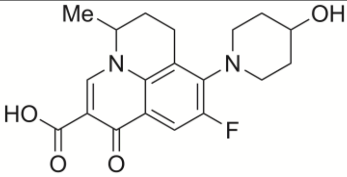 | HTS against *M. tuberculosis* in 7H9 media | inconclusive |
| Naproxen | 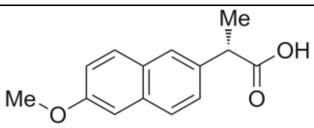 | HTS against H37Rv strain | inactive |
| Naratriptan | 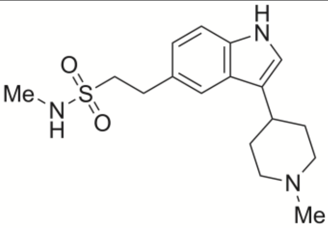 | no | not previously determined |
| Nelarabine | 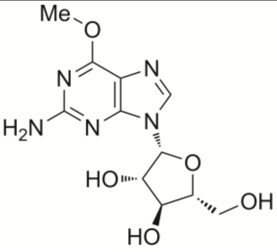 | no | not previously determined |

molecules

MDPI

| | | | |
|---|---|---|---|
| Niclosamide | 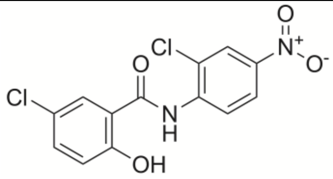 | HTS against H37Rv strain | active |
| Nintedanib | 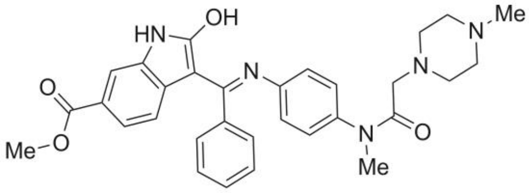 | no | not previously determined |
| Nizoral | 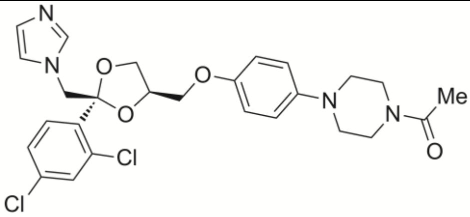 | HTS against H37Rv strain | active |
| Ofloxacina | 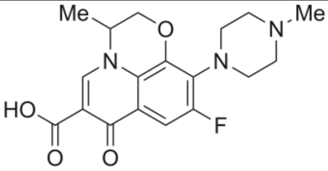 | HTS against H37Rv strain | active |
| Olopatadine | 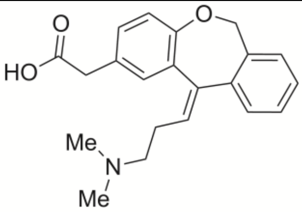 | HTS against H37Rv strain | inactive |
| Pazopanib | 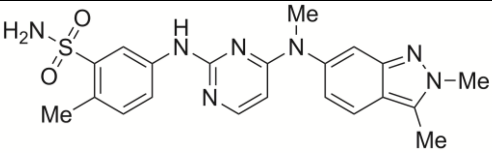 | no | not previously determined |
| Pemetrexed | 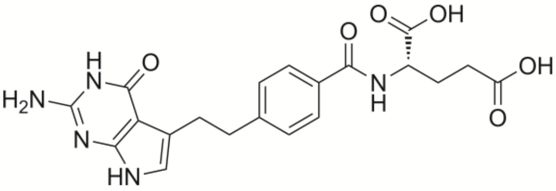 | no | not previously determined |
| Peramivir | 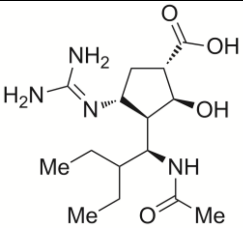 | no | not previously determined |

| | | | |
|---|---|---|---|
| Picosulfuric acid | 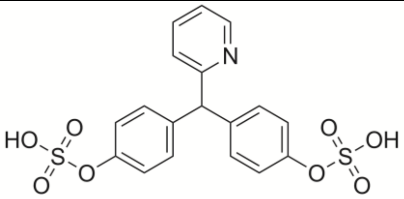 | no | not previously determined |
| Pidolate | 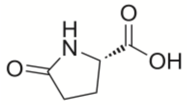 | HTS against *M. tuberculosis* in 7H9 media | inactive |
| Pidotimod | 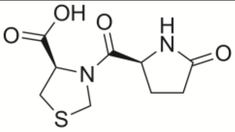 | HTS against H37Rv strain | inactive |
| Pitavastatin | 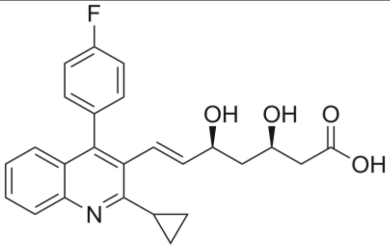 | no | not previously determined |
| Prochlorperazine | 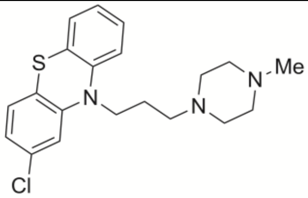 | HTS against H37Rv strain | active |
| Pyritinol | 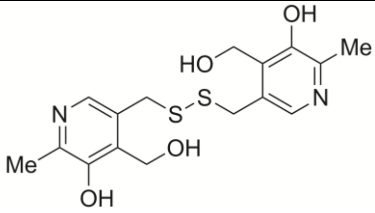 | no | not previously determined |
| Ranelic acid | 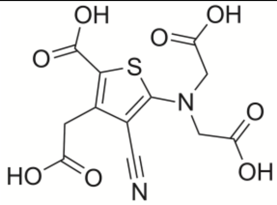 | no | not previously determined |
| Rebamipide* | 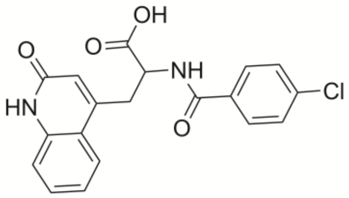 | no | not previously determined |
| Rivabirin | 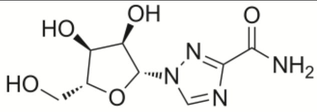 | HTS against H37Rv strain | inactive |

| | | | |
|---|---|---|---|
| Sertraline | 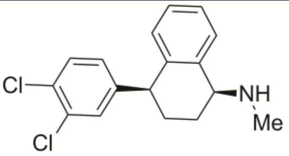 | HTS against H37Rv strain | active |
| Sitagliptin | 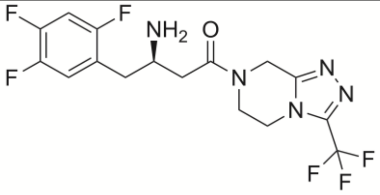 | no | not previously determined |
| Sofalcone | 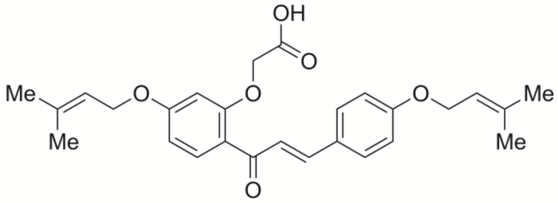 | no | not previously determined |
| Sulfasalazine | 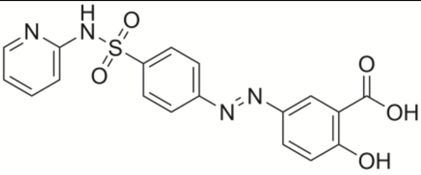 | HTS against H37Rv strain | inactive |
| Sulindac | 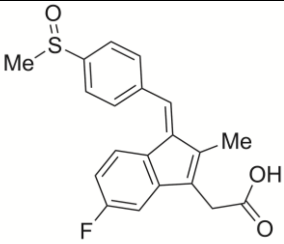 | HTS against H37Rv strain | inactive |
| Tamsulosin | 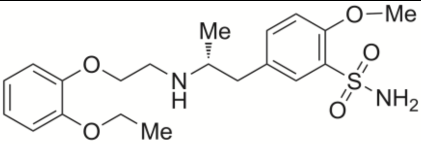 | no | not previously determined |
| Telmisartan | 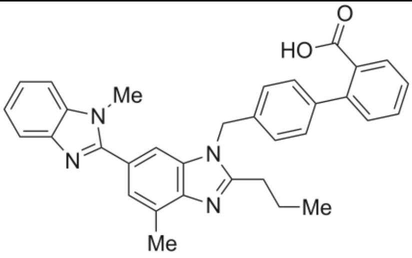 | HTS against *M. tuberculosis* in 7H9 media<br>HTS against H37Rv strain | inactive |
| Terfenadine | 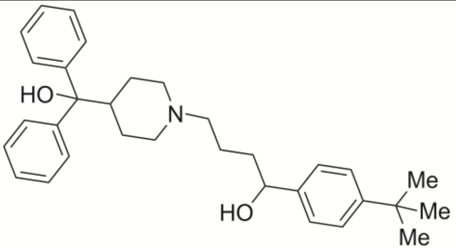 | HTS against H37Rv strain | active |

molecules

| | | | |
|---|---|---|---|
| Terazosin | 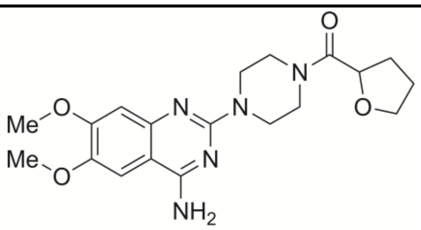 | HTS against H37Rv strain | inactive |
| Thiamphenicol | 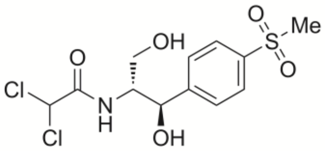 | HTS against H37Rv strain | active |
| Thymopentin | 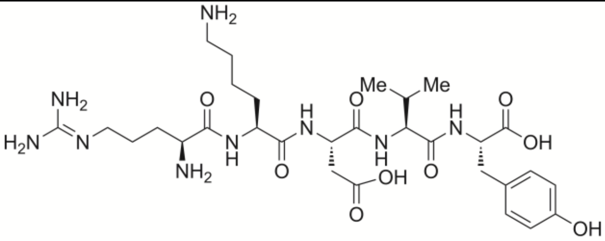 | HTS against *M. tuberculosis* in 7H9 media | inactive |

* These compounds showed high scores against Zmp1 only. Not purchased: compounds that were not available during the work or were available in a small amount for the tests. The data about the *in vitro* tests against *Mycobacterium* were found in PubChem (https://pubchem.ncbi.nlm.nih.gov/). The tests considered were against *M. tuberculosis* H37Rv strain (High Throughput Screen to Identify Inhibitors of *Mycobacterium tuberculosis* H37Rv AID: 1332 https://pubchem.ncbi.nlm.nih.gov/bioassay/1332; AID: 1626 https://pubchem.ncbi.nlm.nih.gov/bioassay/1626) and against *M. tuberculosis* in 7H9 media (High Throughput Screening Assay used to Identify Novel Compounds that Inhibit *Mycobacterium tuberculosis* in 7H9 Media AID: 449762 https://pubchem.ncbi.nlm.nih.gov/bioassay/449762).
